# Short duration waveforms recorded extracellularly from freely moving rats are representative of axonal activity

**DOI:** 10.3389/fncir.2013.00181

**Published:** 2013-11-18

**Authors:** Ashlee A. Robbins, Steven E. Fox, Gregory L. Holmes, Rod C. Scott, Jeremy M. Barry

**Affiliations:** ^1^Department of Neurology, Geisel School of Medicine at DartmouthLebanon, NH, USA; ^2^Program in Experimental and Molecular Medicine, Geisel School of Medicine at DartmouthLebanon, NH, USA; ^3^Department of Physiology and Pharmacology, State University of New York, Downstate Medical CenterBrooklyn, NY, USA; ^4^The Robert F. Furchgott Center for Neural and Behavioral Science, State University of New York, Downstate Medical CenterBrooklyn, NY, USA; ^5^Epilepsy and Development Group, Department of Neurological Sciences, University of Vermont College of MedicineBurlington, VT, USA; ^6^Institute of Child Health, University College LondonLondon, UK

**Keywords:** axonal activity, short duration waveform, *in vivo* electrophysiology

## Abstract

While extracellular somatic action potentials from freely moving rats have been well characterized, axonal activity has not. We report direct extracellular tetrode recordings of putative axons whose principal feature is a short duration waveform (SDW) with an average peak-trough length less than 179 μs. While SDW recordings using tetrodes have previously been treated as questionable or classified as cells, we hypothesize that they are representative of axonal activity. These waveforms have significantly shorter duration than somatic action potentials, are triphasic and are therefore similar to classic descriptions of microelectrode recordings in white matter and of in vitro action potential propagation along axons. We describe SDWs recorded from pure white-matter tracts including the alveus and corpus callosum. Recordings of several SDWs in the alveus exhibit grid-like firing patterns suggesting these axons carry spatial information from entorhinal cortical neurons. Finally, we locally injected the GABA_A_ agonist Muscimol into layer CA1 of the hippocampus while simultaneously recording somatic activity and SDWs on the same tetrodes. The persistent activity of SDWs during Muscimol inactivation of somatic action potentials indicates that SDWs are representative of action potential propagation along axons projecting from more distal somata. This characterization is important as it illustrates the dangers of exclusively using spike duration as the sole determinant of unit type, particularly in the case of interneurons whose peak-trough times overlap with SDWs. It may also allow future studies to explore how axonal projections from disparate brain regions integrate spatial information in the hippocampus, and provide a basis for studying the effects of pharmaceutical agents on signal transmission in axons, and ultimately to aid in defining the potential role of axons in cognition.

## INTRODUCTION

The in vivo firing properties of extracellularly recorded hippocampal pyramidal cells and interneurons (INT) have been well characterized (Ranck, 1973; Fox and Ranck, 1975, 1981; Henze et al., 2000). Reliable identification of these cell types was essential for the experiments confirming their importance in describing the neural systems underpinning spatial cognition and attention (Kentros et al., 2004; Muzzio et al., 2009; Fenton et al., 2010) and in the generation of network oscillations (Kamondi et al., 1998; Penttonen et al., 1998; Buzsaki, 2002; Colgin and Moser, 2010). Apart from activity at cell bodies, normal neural function and therefore properly ordered cognition, requires signaling between neurons and their downstream targets along axons (Swadlow et al., 1980; Debanne, 2004; Womelsdorf and Fries, 2007; Kleen et al., 2010; Singer, 2011; Zalesky et al., 2011). Although the axon has historically been described merely as a reliable conduit for ordered signal propagation, recent experimental and theoretical data have demonstrated that the axon may be directly involved in complex information processing (Debanne, 2004; Bakkum et al., 2013), contribute to high frequency network oscillations (Traub et al., 2003; Dugladze et al., 2012; Scheffer-Teixeira et al., 2012) and possess intrinsic braking mechanisms that can potentially halt seizure propagation (Meeks et al., 2005). Being able to reliably record waveform activity from axons will provide the opportunity to explore these mechanisms in vivo. While great strides have been made in the diffusion weighted imaging of axonal processes (Basser and Pierpaoli, 1996) as well as the visualization of axonal projections (Chung et al., 2013), in vivo axonal activity, as recorded in freely moving animals, has received little attention since early microelectrode recordings were developed (Amassian et al., 1961; Cooper et al., 1969) and remain poorly characterized.

Here we describe tetrode recordings of putative fibers that likely represent extracellular axonal activity in freely moving rats. This unit type is intuitively different from the typical somatic action potentials as evinced by its waveform properties: its defining feature of a significantly shorter peak-trough duration, a triphasic shape, and having its principle activity on only one wire of a tetrode. In addition, these short duration waveforms (SDWs) are found in pure white-matter tracts including the alveus and corpus callosum in the absence of neuronal cell bodies or recorded somatic activity.

Finally, we successfully differentiated axonal and somatic activity in CA1 of the hippocampus using the GABA_A_agonist Muscimol. We further discuss our justifications for SDW classification with regard to similar reports in the literature.

## MATERIALS AND METHODS

### SUBJECTS

Six adult male Long Evans rats were used for recordings in the hippocampus. An additional six adult male Sprague Dawley rats were used for recordings from white matter, the alveus and the region of the corpus callosum. Rats were food deprived within 85% of their pre-deprivation body weight and trained to chase sugar pellets that dropped randomly from an overhead feeder every 30s (see below). All procedures were approved by local institutional animal care and use committee and conducted in accordance with guidelines from the National Institutes of Health.

### SURGERY

Rats were anesthetized with inhaled isoflurane or injected pentobarbital (50 mg/kg i.p.) and placed in a stereotaxic frame. The skull was exposed and four screws inserted, two anterior to the left and right ends of bregma and two left and right over the cerebellum. Grounding was achieved via the right cerebellar screw.

For hippocampus recordings, rats were chronically prepared with an implant (described in detail in Barry et al., 2012; manufactured in the Muller laboratory at State University of New York, Downstate Medical Center, Brooklyn, NY, USA) that allowed for the local injection of pharmacological agents into the hippocampus while recording from a 2 × 4 array of tetrodes. All tetrodes were made from 25 μm diameter nichrome wire, twisted, and cut square. A 22 gage injection guide cannula reached from the top of the implant to 1.8 mm beyond its base. One array of four electrodes was aligned 1.5 mm from the injection site while the remaining linear array of tetrodes approached to 2.0 mm from the injection site. The injection guide cannula was inserted in the rat brain through an opening in the skull (0.8 mm in diameter centered at -3.5 AP and +3.7 ML, above the left dorsal hippocampus) allowing insertion of a cannula for local drug injection. The guide cannula was set at a brain depth of 1.8 mm DV and kept open with 30 gage wire that reached 3.6 mm DV. A 2.6 mm hole 0.5 mm medial to the left guide hole was made for implantation of a 2 × 4 array of tetrodes spaced 0.5 mm apart. The tips of these tetrodes were placed 2.0 mm below the skull surface.

For white matter recordings, data were collected from two animals using the same implants and co-ordinates for hippocampus recordings described above but from more superficial electrodes in the alveus (~2.4 mm DV). For four more animals the white matter recordings were carried using a 2 × 2 array of tetrodes spaced at 0.5 mm intervals. For these four rats, a 2.1 mm hole was made +4.1 mm AP. For two of these rats, the hole was -1.0 mm ML and the implant set at 15^°^. For the remaining two rats, the hole was drilled at -0.5 mm ML and the implant set at 0^°^. Electrodes extended to 2.0 mm beneath the skull surface. For all implants, each tetrode wire was gold plated before implantation until the impedance was between 80 and 130 kOhms.

All implants were fixed to the skull via the skull screws and Grip Cement (Dentsply). The wound was sutured and topical antibiotic applied. The interval between surgery and the beginning of the cell screening process was 1 week.

### ELECTROPHYSIOLOGY AND RECORDING APPARATUS

Methods for training, tracking, electrophysiological recording, and cell screening were similar to Barry and Muller (2011). The rats were tethered to a recording cable while they foraged for sugar pellets in either a square (76 cm × 76 cm) or a small circular (48 cm diameter) arena. Signals from the brain were pre-amplified X1 at the headstage and channeled through the tether cable to the signal amplifiers and computer interface. Signals were sampled at 33 kHz and filtered at 300–6000 Hz (Neuralynx, MT, USA) and EEG signals were recorded from one wire of a tetrode in layer stratum oriens of the hippocampus. This signal was referenced against the ground screw placed above the cerebellum.

The rat’s location in the arena was sampled at 30 Hz (Neuralynx, MT, USA). The activity of individual units was separated offline into different clusters based on their waveform properties (Offline Sorter, Plexon, Dallas, TX, USA). Units and tracking data were then displayed in firing rate maps by dividing the number of spikes in a location by the time in that location (Muller et al., 1987). The relationship between spike and LFP data was used to generate phase maps to show the average phase of unit firing with respect to the theta signal as a function of location.

Each recording day, the animals were placed into the recording apparatus, allowed to explore the arena, and chase sugar pellets that fell from an overhead feeder every 30 s. If no units could be isolated the tetrode drives were lowered. In the case of hippocampal electrode placements, the electrodes were advanced until the activity of approximately 10 pyramidal cells could be isolated. In the case of white matter placements, recordings were carried out for four successive days or until tetrodes displayed SDW units.

### OFFLINE SORTING OF UNIT CLUSTERS

Waveform properties were defined in three-dimensional feature space (Offline Sorter, Plexon, Dallas, TX, USA) by first comparing peak amplitude across all four wires of the recording tetrode. Further processing was performed by using the combination of three additional features, the waveform projection onto the first principal component (PC1), the waveform voltage at any possible hyperpolarization taking place before depolarization (Slice 1), as well as the hyperpolarization following the depolarization (Slice 2).

(1) PC1 = Σp1(*t*)*w(*t*)
(2) Slice 1 = *w*(*i*) :the waveform voltage at time *t* = *i*
(3) Slice 2 = *w*(*i*) :the waveform voltage at a second time point at time *t* = *i*

Where *w*(*t*) = [*w*(1), …, *w*(*n*)] is the waveform (*n* = number of points in a waveform), and p1(*t*) = [p1(1), …, p1(*n*)] is the first principal component vector.

### FIRING RATE MAPS

The raw data recordings comprised of a series of action potential time stamps for each isolated cluster and a 30 Hz series of time stamped *x* and *y* co-ordinates for tracking LEDs mounted on the preamplifier connected to the implant. A 64 by 64 element firing rate array for each cell cluster was first constructed (custom software); each element corresponded to a square pixel ~3.0 cm on a side. To make the spatial firing rate array, the number of spikes for each cell was counted for each element. The total time spent by the animal was then accumulated for each element.

For each cell, the spike array was divided on an element by element basis to form the rate array (Muller et al., 1987). In firing rate maps, yellow pixels represent regions in which the firing rate was exactly zero. Increasing firing rates are represented in the color order: orange, red, green, blue, purple. The number key for each map shows the median firing rate for each color category. Pixels never visited by the rat are white.

Spike amplitude was also measured as a factor of signal to noise ratio using custom software. Signal to noise ratio (S/N) is measured as the average peak to trough amplitude of the spike on the tetrode wire where it is largest divided by the range of the 95% confidence limits on the noise.

### MUSCIMOL INJECTION

The method of Muscimol injection was similar to that previously described by Barry et al. (2012). Briefly, pharmacological agents were injected into the hippocampus of freely moving rats via the guide cannula set in the recording implant prior to rats being placed in the arena.

In order to dissociate the activity of putative axons and cell bodies, the GABA_A_ receptor agonist Muscimol (5-aminomethyl-3-hydroxyisoxazole; Sigma, St. Louis, MO, USA) was injected into the left hippocampus while the activity of several units was recorded in the left hippocampus. Muscimol was used to suppress somatic activity in the hippocampus (Hafting et al., 2008; Barry et al., 2012). If SDWs represent the propagation of action potentials propagating in axons, they should not be directly inactivated by local Muscimol. While GABA_A_ receptors are found on multiple axonal compartments of hippocampal pyramidal cells (Brunig et al., 2002; Trigo et al., 2008; Debanne et al., 2011), activation of GABA_A_ receptors along the axon with GABA or Muscimol, at least in the case of mossy fibers, has varying effects on axonal action currents in vitro dependent on chloride levels (Ruiz et al., 2003). Similarly, Dugladze et al. (2012) recently recorded the activity from both the soma and axon of pyramidal cells in the hippocampus and showed that activating GABA_A_ receptors in the axon did not inhibit the propagation of orthodromic action potentials. These results suggest that if one were to record the activity of an individual axon, as well as the activity of somatic action potentials in the presence of Muscimol, that the action potentials in the axon may not be inactivated. However, the persistence of axonal activity following Muscimol exposure may vary relative to the distance between the somatic source of the axonal activity and the drug injection site. If the drug injection site were close to the soma, axonal activity would appear to inactivate at the same time as the simultaneously recorded somatic activity. To this end, diffusion of Muscimol through the hippocampus will be taken into account here.

We simultaneously recorded the unit activity of hippocampal cell bodies and putative axons, whose extracellular waveforms are described below, on the same tetrodes before and after local injections of Muscimol in order to inactivate somatic activity (Hafting et al., 2008; Barry et al., 2012). Muscimol (0.5 μg/μl in phosphate buffered saline) was infused into the left hippocampus (1.0 μl at 0.33 μl/min) of six rats via a 30 gage injection cannula that entered the brain through the surgically implanted guide cannula. The injection cannula was withdrawn 3 min later. Approximately 5 min following the start of the injection, the rat was returned to the recording box for a 45–60 min recording session. Unit activity was separated by tetrode for each rat and only tetrodes that included both SDW activity and longer duration somatic activity were included in analysis. Data from three/six rats included somatic activity and SDWs from multiple tetrodes that were either 1.5 or 2.0 mm from the injection site. These data allow for a description of the effect of Muscimol injection on the activity of SDWs and cell bodies over time as Muscimol diffuses through the hippocampus.

The overall firing activity of individual units was calculated over 3 min epochs during both a 15 min pre-injection baseline and the post-injection recording session. The firing activity was then normalized by the first 3 min epoch of the baseline recording sessions. Units with an overall firing rate <0.1 Hz over the first 3 min epoch were excluded from analysis. In the post-injection period, units were considered to be inactivated if their individual or averaged overall firing rate was less than or equal to 5% of their baseline firing rate.

### HISTOLOGY

Brains from rats used for white matter recordings were sliced at 20 μm thickness using a cryostat before being placed directly onto slides. The slices were then stained with either thionin, DAPI (Dapi Fluropure, #D21490; Invitrogen, Eugene, OR, USA) or DiI (#D282; Invitrogen, Eugene, OR, USA) in order to visualize the electrode tracks.

## RESULTS

**FIGURE 1:**
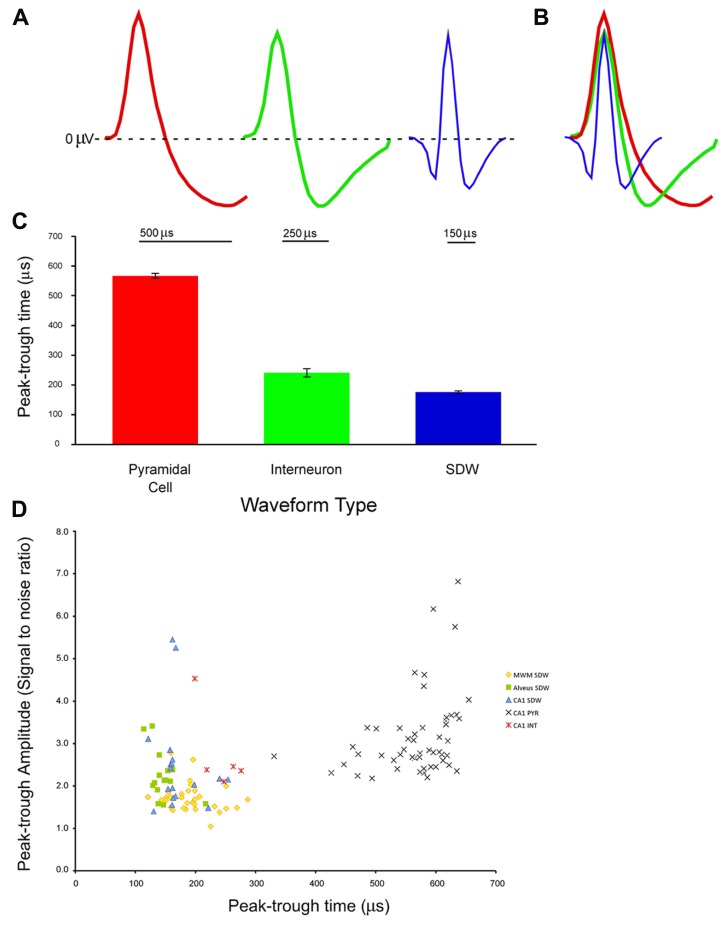
**Example of waveforms from extracellular tetrode recordings in the hippocampus from different cell types and a putative axon: (A)** pyramidal cell, interneuron, and short duration waveform. The pyramidal cell (red) has an average spike width of 500 μs with a long lasting hyperpolarization. The interneuron (green) has a shorter spike width of 250 μs with a shorter hyperpolarization period. The third example is of a short duration waveform that is triphasic, exhibiting a brief hyperpolarization period before and after a brief depolarization; **(B)** overlay of the three average waveforms; **(C)** average and standard error of peak-trough time for pyramidal cells (*n* = 53, 567 ± 8.68 μs), interneurons (*n* = 5, 241 ± 14.1 μs), and putative axons (*n* = 64, 176 ± 4.84 μs) recorded in the medial white matter (MWM), alveus, and pyramidal cell layer of the hippocampus; **(D)** Scatter plot of signal to noise ratios for individual units against peak-trough time for SDWs recorded in MWM, alveus, and CA1 of the hippocampus, as well as pyramidal cells (PYR) and interneurons (INT) in CA1 of the hippocampus. The SDWs, and pyramidal cells clearly cluster by peak-trough time while interneurons overlap with the longer SDWs.

Recordings made from tetrodes positioned in the CA1 cell layer in 6 rats yielded 53 putative pyramidal cell units having a mean peak-trough duration of 567 ± 8.7 μs (Average S/N = 3.14 ± 0.13), 5 putative INT having a mean spike duration of 241 ± 14.1 μs (Average S/N = 2.8 ± 0.5), and 18 SDWs having a mean spike duration of 172 ± 8.2 μs (Average S/N = 2.5 ± 0.3). Recordings from tetrodes in the medial white matter from 4 rats yielded 31 SDWs with a mean peak-trough duration of 193 ± 6.9 μs (Average S/N = 1.7 ± 0.1). Finally, from tetrode recordings in the alveus from 2 rats, 15 SDWs were found with a mean peak-trough duration of 146 ± 6.01 μs (Average S/N = 2.2 ± 0.15). Recordings made in both white matter regions were made in the absence of somatic action potentials. Example waveforms, the average peak-trough duration and standard error for each cell type as well as all SDWs are shown in **Figures 1A–C**). A scatterplot comparing the amplitude (S/N ratio) and peak-trough duration (μs) of each putative cell and SDW is shown in **Figure 1D**.

All SDWs were found to have triphasic extracellularly recorded action potentials, i.e., they exhibited a brief period of positivity before and after the negative spike associated with their local depolarizing phase. In addition, the voltage changes are largely on only one wire of a tetrode for SDWs. In contrast, none of the waveforms typically identified as representing INT exhibited positivities prior to their negative spikes and they generally exhibited similar magnitude voltage changes on multiple wires of a tetrode.

### SDW VARIATION BY REGION

An ANOVA comparing the peak-trough duration of SDWs recorded from the medial white matter, alveus, and CA1 of the hippocampus indicates that not all SDWs share a common range of peak-trough duration (*F*_2,61_ = 9.46, *p* = 2.60 × 10^-^^4^). SDWs from alveus have the shortest peak-trough duration while those found in the medial white matter and pyramidal layer of CA1 are not significantly different from each other (*t*_47_ = 1.90, *p* = 0.063).

An ANOVA comparing the S/N values of SDWs in each region suggests that SDWs recorded in the MWM tended to be smaller in amplitude than those recorded in the alveus or layer CA1 (*F*_2,61_ = 7.46, *p* = 0.001). The S/N values for SDWs in the alveus and CA1 were similar (*t*_31_ = 0.8, *p* = 0.43).

### CELL TYPES AND SDWS ARE DISTINCT BY DURATION

An ANOVA reveals all 64 SDWs, with a mean peak-trough duration of 176 ± 4.9 μs, were significantly different from both types of somatic activity recorded in the CA1 cell layer of the hippocampus (*F*_2,119_ = 871.62, *p* = 8.47 × 10^-^^72^). All three unit types are significantly different from each other (**Figure 1C**) with pyramidal cells having the longest peak-trough duration. This finding is reinforced by a scatterplot for each for each recorded cell type and SDW recording location is plotted by amplitude (AD units) and peak-trough duration (μs; **Figure 1D**). The SDWs cluster toward the left while the pyramidal cells cluster toward the right. Importantly, INT overlap somewhat with the longer duration SDWs. In addition, many of the SDWs were similar in amplitude to the somatic action potentials with the largest amplitude SDWs in the alveus or medial white matter.

### SPATIAL FIRING PATTERNS OF SDWS

**FIGURE 2:**
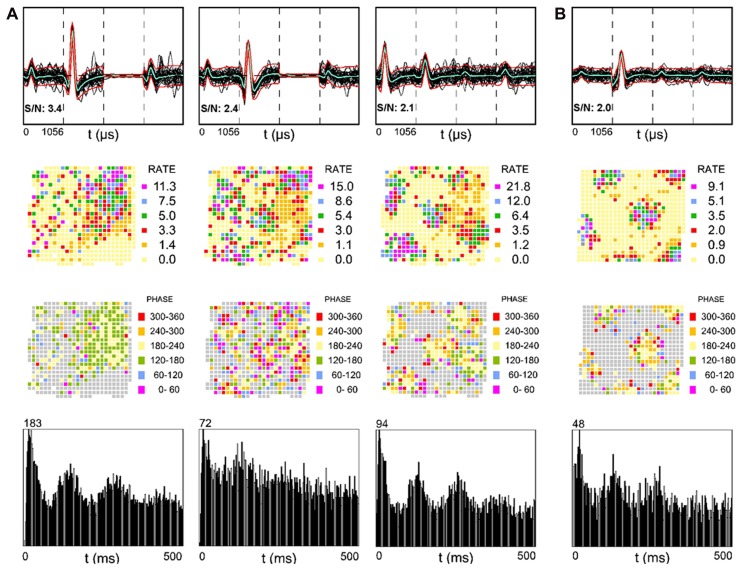
**Examples of SDWs recorded from the alveus in the absence of somatic activity compared across each wire of a tetrode (top row) and its corresponding firing rate map (second row), phase map (third row), and autocorrelogram (fourth row).** The three examples on the left were simultaneously recorded from one rat **(A)** while the example on the far right was recorded from a different rat **(B)**. The overlay of 60 spikes as well as the average waveform for each SDW is displayed for each wire (top row). The third channel in each of the two examples on the far left is flat, denoting a malfunctioning electrode channel. recording channel. The discharges of several of the SDWs shown are indicative of grid patterns (second row) and appear to be organized by local theta oscillations (third row) as spike activity advances from late to early phases of the theta cycle as the animal passes through each grid vertex. Averaged across the entire 16 min session, the spiking of the SDWs on entry into the periphery of each field tends to occur on one phase, while the firing in the central part of each field tends to be earlier by about 180^°^. The firing pattern the first putative axon in 5a resembles a place field but is phase locked between 120 and 240^°^. The bottom row shows the autocorrelation for each putative axon and is indicative of significant theta modulation in their firing activity.

The alveus is a border structure composed of axons. Some of these axons carry efferent output from pyramidal cells toward the fimbria/fornix and the adjacent retrohippocampal areas and others carry afferent input from more distant brain regions such as the entorhinal cortex (Deller et al., 1996; Brun et al., 2008). **Figure 2A** shows three simultaneously recorded SDW units from one rat, while **Figure 2B** shows a single SDW unit recorded from a second rat. The activity of three of the four putative axons shown have a triangular array of firing fields (**Figure 2**, second row) resembling those typical of grid cells (Hafting et al., 2005; Boccara et al., 2010) that could be projections from the entorhinal cortex, presubiculum, or parasubiculum (Hafting et al., 2005; Boccara et al., 2010). All of the grid-like units recorded are shown in **Figure 2**. Grid cells from layer II of the MEC show phase precession (O’Keefe and Recce, 1993; Huxter et al., 2003) in that their spike activity advances from late to early phases of the theta cycle as the animal passes through a grid vertex (Hafting et al., 2008). A similar organization of SDW spikes by theta oscillations is also present in the alveus (**Figure 2**, third row). Averaged across the entire 16 min session, the spiking of the SDWs while the rat is in the central part of each field tends to occur between 180 and 240^°^ of the theta cycle while firing at the periphery of each field tends to be greater or less than that range in the theta cycle. In contrast to the other three putative axons illustrated, the leftmost firing rate map in **Figure 2** resembles a place field but shows no evidence of phase precession. This SDW appears to fire mainly between 120 and 240^°^. The autocorrelograms at the bottom of **Figure 2** suggests that these putative axons are also strongly theta modulated.

**FIGURE 3:**
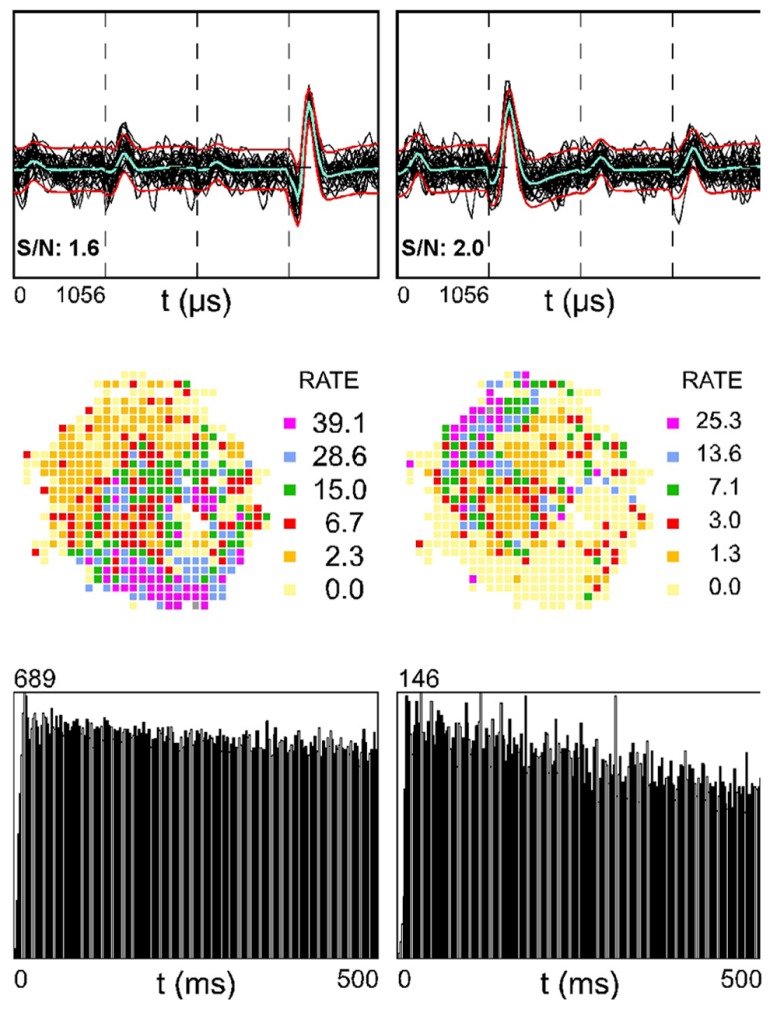
**Two simultaneously recorded SDWs in the medial white matter in the absence of somatic activity compared across each wire of a tetrode (top row) and its corresponding firing rate map (second row) and autocorrelogram (third row).** The overlay of 60 spikes as well as the average waveform for each SDW is displayed for each wire (top row). The firing rate maps for the SDWs display a complimentary spatial firing pattern where the SDW on the left fires more when the SDW on the right fires at a low rate (second row). The autocorrelograms for each SDW (third row) suggest that they are not theta modulated.

**Figure 3** shows two simultaneously recorded SDWs (top row) in medial white matter. The rate maps of these putative axons appear to be complementary (middle row). That is, the firing activity of the SDW on the left tends to be slower in the region of the place field for the unit on the right. If the two fields were superimposed, they would create a rate map with high firing rate covering the entire arena. The autocorrelation (bottom row) indicates that neither unit is theta modulated.

**FIGURE 4:**
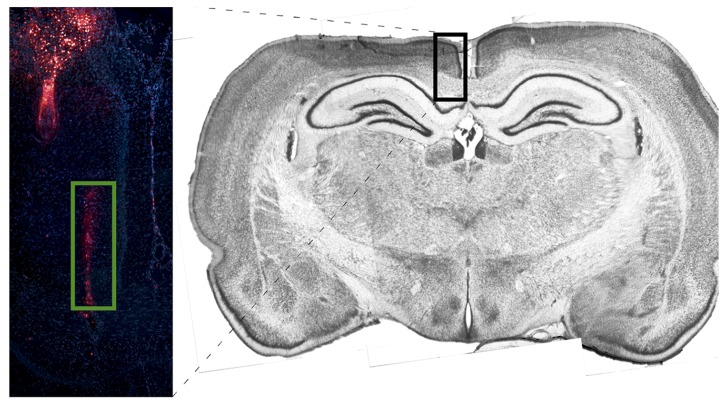
**Image depicting probe placement for one corpus callosum animal.** Electrodes were coated with DiI and brain slices were stained using DAPI. One probe is shown to the left of midline with its lowest point in the center of the corpus callosum. The DiI stain is compared against a coronal section (~4.2 mm from Bregma) taken from a non-implanted rat of the same strain and age stained with Thionin.

**Figure 4** depicts tetrode placement in white matter, near the corpus callosum. Electrodes were coated with DiI and brain slices were stained using DAPI. One probe is shown to the left of midline with its lowest point toward the center of the corpus callosum.

### PHARMACOLOGICAL SEPARATION OF SOMATIC AND AXONAL ACTIVITY IN CA1

**FIGURE 5:**
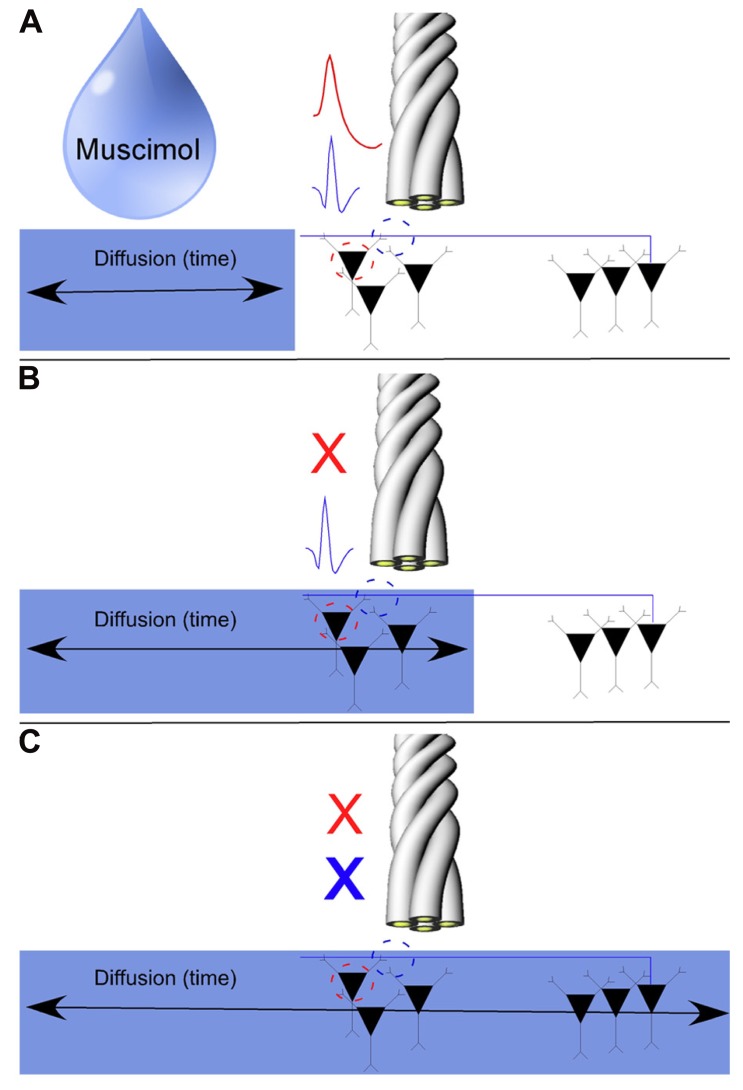
**Model for the pharmacological separation of putative axonal activity from somatic activity using the GABA_A_** agonist Muscimol. As axons tend to have a sparse density of GABA_A_ receptors, axonal activity should not be inactivated by local injections of Muscimol. We record somatic action potentials and SDWs simultaneously on the same tetrode **(A)**. Somatic action potentials are inactivated over time as Muscimol diffuses through the hippocampus **(B)**. If SDWs are truly representative of action potential propagation along axons (example SDW shown in blue), then Muscimol should inactivate local somatic activity when it diffuses to the recording site (example pyramidal waveform shown in red) while local SDW activity should persist **(B)** until sufficient diffusion time has elapsed for Muscimol to inactivate the distal somatic source of the axonal action potential **(C)**.

Our results to this point have been largely descriptive. The inherent waveform properties of SDWs as well the fact that they are found in white matter in the absence of somatic activity, are supportive of our notion that SDWs are representative of axonal rather than somatic activity. As Muscimol inactivates somatic activity in the hippocampus (Hafting et al., 2008; Barry et al., 2012) and GABA_A_receptors are sparse along the extent of the axon (Brunig et al., 2002; Trigo et al., 2008), we used Muscimol to separate the somatic activity from the putative axonal activity. In short, local injections of Muscimol silenced somatic action potentials while simultaneously recorded SDWs on the same tetrodes remained active. The SDWs would then typically remain active until the Muscimol diffused to their more distal somatic source (see **Figure 5** for a detailed description of the model). As there cannot be any locally generated axonal activity if Muscimol silences all local somatic activity from pyramidal cells and INT, any SDWs remaining after Muscimol must come from axons whose cell bodies are beyond the range of the Muscimol diffusion.

We compared the firing activity of 58 putative CA1 somatic units to the activity of 18 SDWs before and after the local injection of Muscimol into the left hippocampus. The amplitude (AD units) and peak-trough duration for all SDWs and somatic units are shown in **Figure 1C**. We compared the average normalized firing frequency of these units during the 3 min epoch when the average somatic activity on each of the tetrodes (*n* = 10) reached 5% or less of baseline rate. Our results show that the impact of Muscimol on unit firing is significantly different for SDWs compared to somatic units. Specifically, a *t*-test reveals that SDWs are significantly more active in the presence of Muscimol, firing at an average of 84 ± 16% of their baseline firing rate (*t*_9_ = -5.12; *p* = 7.23 × 10^-^^5^), when hippocampal pyramidal cells were firing at an average 3.3 ± 0.62% of their baseline rate.

**FIGURE 6:**
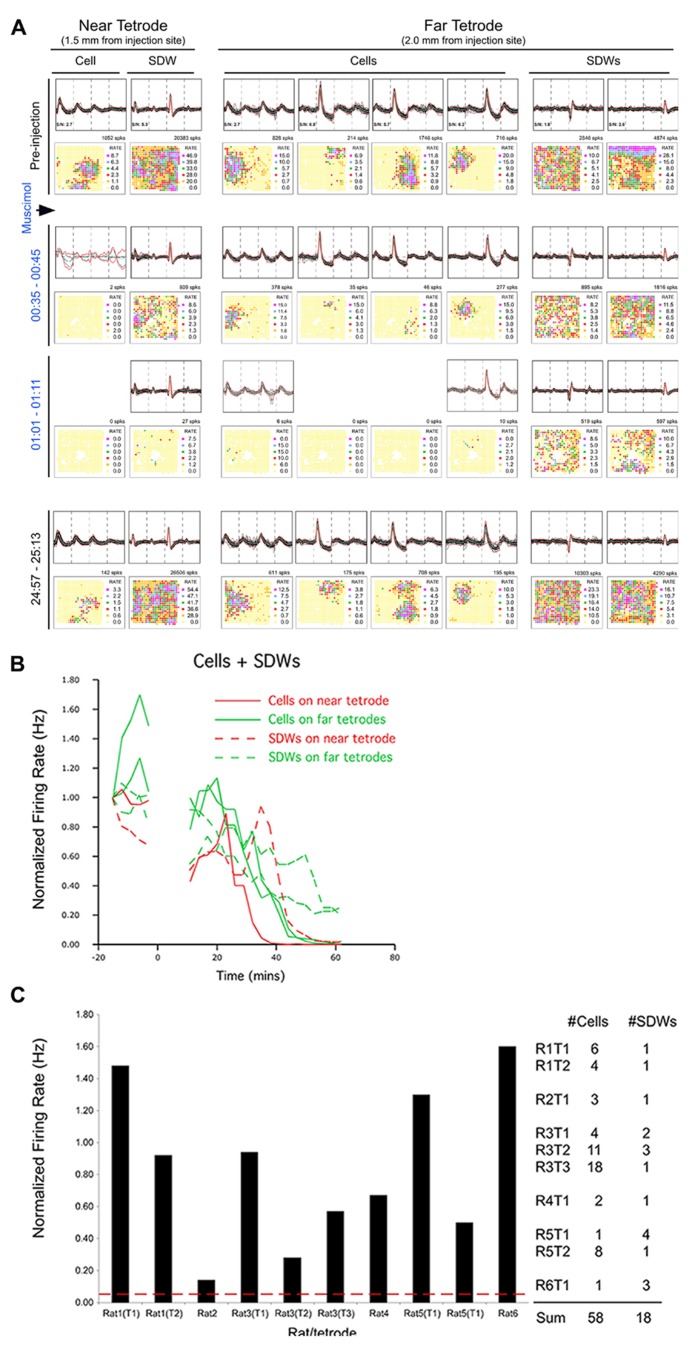
**Pharmacological separation of simultaneously recorded SDWs and somatic activity following local Muscimol injection.** Somatic activity is inactivated my Muscimol while putative axonal activity persists. **(A)** An example of the separation of axonal and somatic activity recorded on the same tetrodes by Muscimol. The top row shows the overlay of several spikes and the average waveform of somatic action potentials as well as the SDWs of putative axons compared across each wire of a tetrode. The examples of the far left were recorded on a tetrode 1.5 mm from the injection site (near tetorde) while those on the right were recorded 2.0 mm from the injection site (far tetrode). One of the SDWs on the far tetrode was triphasic but inverted, suggesting it was inside or very close to a putative axon. This was the only case of inversion found from all SDWs. The second row displays the firing rate maps for each unit type shown above as well as the number of spikes on the upper right. The somatic action potentials were found to have clear firing fields while the SDWs did not. The waveforms, as well as firing rate maps are then shown for the same units for 10 min recording sessions 35 min, 1, and 25 h post injection. Waveforms identified as somatic action potentials were silenced before SDWs. The SDW on the near tetrode was inactivated at the same time as somatic action potentials on the far tetrode. This implies that the somatic source of the axonal activity, as represented by the SDW, was approximately 0.5 mm away. Recordings made from the same units 25 h after Musicmol injection (bottom row) shows the reinstatement of all unit activity following the clearance of Muscimol. SDWs therefore tend to be as stable as somatic action potentials; **(B)** Line Plots of the averaged normalized firing rates as a function of time for somatic action potentials and SDWs on near and far tetrodes for the example shown in **(A)**. Cells on the far tetrode become silent after cells on the near tetrode as a product of Muscimol diffusion time. Similar time courses are seen for the SDWs although their activity is prolonged on both near and far tetrodes compared to cells. Note that SDW activity on the far tetrodes continues beyond 60 min after the Muscimol injection; **(C)** average normalized overall firing rate for SDWs from each rat and tetrode during the 3-min epoch when simultaneously recorded somatic activity dropped to at least 5% of baseline levels. The number of cells and SDWs from each rat and tetrode are indicated on the right. The vast majority of SDWs are significantly active while the somatic activity recorded from the same tetrodes has been silenced by Muscimol.

In a few cases, SDW activity slowed during the epoch of somatic inactivation and, with time, SDWs became inactive. An example is shown in **Figure 6A** from a putative axon recorded on the tetrode positioned closer to the injection site (near tetrode). Muscimol-induced inactivation of somatic waveforms occurs first on the near tetrode and, with the diffusion of Muscimol over several minutes, occurs in somatic waveforms at the far tetrode (0.5 mm between tetrodes). The near tetrode SDW was inactivated concurrently with the somatic waveforms on the far bank. This implies that the Muscimol inactivated the somatic source of the SDW activity 0.5 mm from its recording site. The SDW activity on the far tetrode, while decreased in frequency, continued until the end of the recording session. This indicates that the cell bodies were further removed from the injection site. The average normalized firing activity for both cell bodies and SDWs in this case, and for both near and far tetrodes, is described in the line plot in **Figure 6B**.

The mean normalized firing rate for SDWs from each rat and tetrode during the 3 min epoch when simultaneously recorded somatic activity fell to <5% of baseline levels is shown in **Figure 6C**. The number of somatic waveforms and SDWs from each rat and tetrode are indicated on the right. The firing rate of SDWs in response to Muscimol was variable, with some putative axons increasing their activity beyond baseline firing rate while most gradually decreased activity over time. While most SDWs continued to fire robustly in the presence of Muscimol, there were two interesting outliers. One SDW did inactivate at the same time as somatic waveforms recorded on the same tetrode. As other simultaneously recorded putative axons remained active, this implies that we are able to record different time courses from different axons on the same tetrode. Furthermore, it suggests that the SDW was from the axon of a nearby cell body. Another SDW fell to 14% of baseline 16 min post injection but fell no further after 53 min of recording.

Our results suggest that the SDWs are representative of the propagation of action potentials along axons that extend from distant cell bodies. Moreover, the results imply that SDWs cannot be somatic action potentials as Muscimol reliably inactivates somatic activity. Finally, axons may show different pharmacodynamics than simultaneously recorded somatic activity as Muscimol may cause brief periods of excitation in the activity of some SDWs (**Figure 6C**), perhaps due to disinhibitory processes via suppression of an intervening interneuron.

## DISCUSSION

We have successfully demonstrated that it is possible to monitor axonal activity in white and gray matter and to simultaneously record ensembles of cells and axons using conventional tetrodes. We describe tetrode recordings of SDWs using chronically implanted tetrodes in awake, freely moving rats. These recordings were made in the gray matter of the hippocampus in layer CA1 or from white matter tracts near the corpus callosum and the alveus. The principal feature of SDWs is brief, triphasic action potentials with a mean peak-trough duration of 176 μs. We have also shown that, in hippocampal gray matter recordings, a local injection of Muscimol near the recording tetrodes inactivates somatic action potentials while many SDWs show no significant change in firing rate, at least in the first couple of minutes after somatic inactivation. We suggest this is because the recorded SDWs are representative of axonal activity projecting from somata more distant from the site of injection and are therefore relatively unaffected by Muscimol. Moreover, the sparse density of axonal GABA_A_ receptors would preclude an effect of Muscimol on the axons themselves. Taken together with the inherent properties of the SDWs that we have described, we strongly suggest that SDWs are representative of axonal activity as opposed to direct somatic activity.

### SDWs REPRESENT AXONAL ACTIVITY

We propose that SDWs represent the propagation of action potentials along axons based on three electrophysiological features: first, SDWs were of extremely short duration. Second, SDWs exhibited three phases (from a brief small positive phase, to a longer-duration negative phase, to another brief small positive phase). Both of these features have been associated with extracellular recordings of action potentials in axons in vitro (Johnston and Wu, 1995; Raastad and Shepherd, 2003; Meeks et al., 2005; Kole et al., 2007; Meeks and Mennerick, 2007; Dugladze et al., 2012). The waveform shape of the axonal action potentials reported by Raastad and Shepherd (2003) are a particularly close match to the examples of SDWs provided here. With regard to spike duration, Kole et al. (2007) found a significant decrease in the length of action potentials from the soma through the extent of the axon in layer five pyramidal cells. The half-width of the action potential decreased significantly as the patch recording sites moved from the soma (503 ± 7.4 μs), the most distal region of the axon initial segment (290 ± 18.8 μs), to axon blebs up to 720 μm from the axon hillock (266 ± 8.5 μs). The first order derivative of axon bleb values match the duration values of extracellularly recorded SDWs (see Henze et al., 2000). Morever, Kole et al., further provide a mechanism for the compression of the spike signal from the soma through the axon. The authors propose that Kv1 channels strategically positioned in the axon initial segment decrease the duration of the axonal action potential waveform and allow for the integration of slow subthreshold signals. In this manner the Kv1 channels are able to control the presynaptic action potential waveform and synaptic coupling in local circuits.

Our description of SDWs also matches that of classic fiber tract recordings using 3 μm diameter tungsten wires set in micropipettes. Both Amassian et al. (1961) and Cooper et al. (1969) reported recordings of brief triphasic action potentials approximately 130 μs in duration in a variety of species (cat, squirrel, squirrel monkey) and recording locations (optic tract, geniculostriate fibers in the visual cortex, pons and medulla, and also the cuneate nucleus). Similar to these reports, SDWs were also found to be quite stable (see 24 h recordings of SDWs in **Figure 6A**). While the largest amplitude axonal spikes reported by Cooper et al. (1969) were ~150 μV, we have recorded several SDWs that were significantly larger in amplitude, particularly in the alveus (see **Figure 1C**).

While we are cautious to generalize the applicability of our peak-trough duration values as criteria for SDW classification in all species and all brain regions, particularly when there is variation between white matter regions, duration in combination with waveform shape should serve as general guidelines for the isolation of *in vivo* axonal activity. In the case of tetrode recordings, activity for SDWs also appears to be typically restricted to a single wire and is therefore suggestive of a much smaller source area as compared to recordings in the area of the soma. This feature could also be added to the short list of SDW criteria.

### BRIEF DURATION WAVEFORMS THAT ARE NOT REPRESENTATIVE OF AXONAL ACTIVITY

An illustration of the dangers of using peak-trough duration as the sole criteria for unit identification can be seen in **Figure 1C**. While the average interneuron peak-trough duration is significantly different from SDWs in each region as well as the average of all SDWs, the INT can overlap with the upper range of SDWs. While the peak trough-duration of the INT in our data set were over 200 μs, Bartho et al. (2004) report putative INT that fall well within the lower range of our SDWs. Assuming that these brief INT were not axonal, it is possible that SDWs could be easily misclassified as INT. Even in early descriptions of putative cortical stellate neuron waveforms, Mountcastle et al. (1969) preferred to refer to their waveforms with the neutral descriptive term “thin spikes” due to the uncertainty that they may have been thalamocortical fibers. As we recorded SDWs in white matter and distinguished between somatic and axonal activity using Muscimol, we are confident that our SDWs were not INT. Moreover, as none of the INT in our data set were triphasic, we suggest that waveform shape, in combination with spike duration, should be a secondary criteria for neuronal characterization.

While the distinction between axon and interneuron peak-trough duration may be sufficiently problematical, there is an additional ongoing debate that pyramidal cells in primate neocortex may have much briefer spikes than in rodents (Vigneswaran et al., 2011). As a consequence, the well established differences between spike durations of INT and pyramidal neurons have now been blurred. Assuming that pyramidal cells that exhibit “thin spikes” are not axons, this finding further points to the pitfalls of using spike duration as a means of globally characterizing neuronal activity.

### REPORTS OF PUTATIVE SDWS

Leutgeb et al. (2000, 2007) have reported head direction units in the hippocampus as well as grid unit in the axon terminals of the perforant path, that may both serve as a source of convergence onto place cells. In the case of Leutgeb et al. (2007), the authors imply that the spikes generating grid patterns may have originated from intact or punctured axons but were hesitant to refer to them as such. Moreover, several of the units shown in Leutgeb et al. (2000) as well as the four units shown in Leutgeb et al. (2007) have similar spike durations and have the same triphasic waveform of the SDWs that we show here. In addition, histology shown in Leutgeb et al. (2000) indicates that several of these head direction units were recorded near white matter tracts in the region of the alveus as well as in the stratum lacunosum-moleculare. In the case of Leutgeb et al. (2007), units displaying grid patterns were recorded in the axon terminals of the entorhinal cortex in the perforant path. Given these similarities, we suggest that the short duration units described in these studies may have been action potentials propagating along axons from other regions of the brain. In addition, the putative grid SDWs we describe here may provide further evidence of such spatial information converging on the hippocampus. As both Leutgeb et al. (2000) and Leutgeb et al. (2007) suggest, the combination of both head direction and grid signals in the hippocampus would allow for strong synaptic interactions which could integrate these spatial processes in the generation of hippocampal place representations. It is our hope that recordings of SDWs in freely moving animals could lead to new ideas regarding the integration of multiple streams of converging spatial information in the generation of the cognitive map (O’Keefe and Nadel, 1978).

## Conflict of Interest Statement

The authors declare that the research was conducted in the absence of any commercial or financial relationships that could be construed as a potential conflict of interest.

## AUTHOR CONTRIBUTIONS

All of the authors contributed to the design of the experiments, interpretation and analysis of the results, and editing of the manuscript. Jeremy M. Barry and Ashlee A. Robbins trained rats, built implants, made recordings, analyzed data, and carried out the experiments. Steven E. Fox provided custom software.
